# Does a tailored magnetic resonance imaging technique affect the surgical planning and outcomes for different cystic urethral and periurethral swellings in females? Seven years tertiary center experience

**DOI:** 10.1007/s00345-022-03973-w

**Published:** 2022-03-16

**Authors:** Wally Mahfouz, Hebatallah Hassan Mamdouh Hassan, Marilena Gubbiotti, Mohamed Elbadry, Ahmed Moussa

**Affiliations:** 1 Urology Department, Alexandria University, Alexandria, Egypt; 2 Radiology Department, Alexandria University, Alexandria, Egypt; 3 Urology Department, San Donato Hospital, Arezzo, Italy; 4 Urology Department, Minia University, Minia, Egypt

**Keywords:** MRI pelvis, Urethral and periurethral cystic swellings, Urethral diverticula, Surgical planning

## Abstract

**Purpose:**

To evaluate the use of magnetic resonance imaging (MRI) in preoperative delineation and surgical planning for the management of female urethral and periurethral cystic vaginal swellings, with emphasis on postoperative surgical outcomes.

**Materials and methods:**

This is a retrospective analysis of females complaining of periurethral and urethral cystic swellings referred to our tertiary center, who underwent MRI for preoperative planning in the period from January 2014 till January 2021, with a total number of 57 patients. Data retrieved from the medical records included: patients’ demographics, presenting symptoms and signs, preoperative radiological investigations, duration of symptoms, previous surgical intervention, detailed intraoperative data, postoperative complications, and postoperative follow-up.

**Results:**

Urethral diverticulum was the commonest cystic lesion representing (64.9%) followed by Skene gland cysts in 14%, Mullerian cysts in 7%, Gartner cysts in 3.5%, and dermoid inclusion cysts in 10.5%. MRI precisely diagnosed the various pathological entities and anatomical complex lesions prior to surgery. This was confirmed after surgery and pathology analysis. All patients were followed up with a mean duration of 35 months, without any evidence of recurrence.

**Conclusion:**

MRI as a standalone imaging technique is mandatory for diagnosis of all urethral and periurethral cystic lesions, as it offers the most accurate diagnostic modality for delineation of these lesions and hence aids in the preoperative surgical planning, aiming to avoid recurrence and improving surgical outcomes.

## Introduction

Female urethral and periurethral cystic swellings are not a common pathology. Presenting symptoms are multiple, including vaginal fullness and discomfort, urinary symptoms including recurrent urinary tract infections (UTIs), urinary incontinence (UI), obstructive urinary symptoms, and discharging pus or urine from urethral meatus. Differentiating these lesions clinically is challenging due to symptoms overlap and difficulty in identifying the origin of these cystic swellings [1].

There is a wide range of differential diagnoses of these cystic lesions, with urethral diverticulum (UD; the most common lesion), Skene duct cyst with classical juxtameatal location, and less commonly anterior vaginal wall cysts such as Mullerian remnant cyst, Gartner cyst, and inclusion dermoid cysts [2].

The incidence of female UD is 1–6% [3], often associated with dysuria, dyspareunia, and postvoiding drippling. The character and complexity of these diverticula together with their ostial communication must be accurately determined pre-operatively to improve post-surgical outcomes.

Different urethral imaging modalities had been utilized to identify the nature of these swellings, including voiding cystourethrogram (VCUG) and double-balloon urethrography (DBU). They allow both delineation of the UDs by contrast and identification of their ostial communication to the urethra. However, they are invasive tools due to need for catheterization, radiation exposure, necessity to void, and technically difficult to perform. All these shortcomings make these modalities equivocal, and thus, additional imaging studies are needed [4].

Various ultrasound (US) techniques (transvaginal, translabial, and endourethral) were compared to VCUG, showing higher sensitivity in detecting the neck of UD compared to contrast modalities. It is also helpful in detecting nearby lesions such as infected periurethral cysts and leiomyomas, which are all not visualized with VCUG. However, US is operator-dependent, and depends on diverticular size and its connection to the urethra [5].

More recently, magnetic resonance imaging (MRI) has become the gold standard modality in the diagnosis of these vaginal swellings. It is non-invasive, with no need for catheter placement. There is no radiation exposure nor contrast injection. MRI gives global spatial configuration of the soft-tissue swellings present with accurate anatomical demarcation of the urethra and the vaginal tissues [6, 7].

The objective of this study was to evaluate the use of MRI in preoperative delineation and surgical planning for the management of different female urethral and periurethral cystic swellings, paying particular attention to the postoperative surgical outcomes.

## Materials and methods

This was a retrospective analysis of the medical records of females with anterior vaginal cystic swellings, being referred to our tertiary center, in the period from January 2014 to January 2021. Local institutional review board and ethics committee (Faculty of Medicine, Alexandria University, Egypt) approval was obtained. Fifty-seven patients were eligible for the study. Data collected were patients’ demographics, presenting symptoms and signs, urogynecological examination data, preoperative radiological investigations performed, duration of symptoms, previous surgical intervention, detailed intraoperative surgical data, post-operative complications, and post-operative follow-up. Preoperative MRI studies were available for all the 57 females included. Out of the 57 females, 13 patients were referred to our center with recurrent vaginal swellings after being operated upon in other hospitals. All these thirteen patients did not perform MRI pelvis prior to their first surgical intervention.

### Imaging technique and surgical procedure

Patients underwent non-contrast MRI pelvis prior to the proposed surgical procedures. MRI was performed on a 1.5 Tesla closed-configuration system Siemens ESPREE (Erlangen, Germany) using a pelvic phased-array coil. Patients were instructed not to urinate for at least 1 h prior to the examination to ensure a semi-full bladder. The patients underwent imaging before and after urination, with adoption of postvoiding sequence to monitor any increase in size of the suspected mass, which was likewise detected live during straining sequence performed before and after voiding. No urethral catheterization was needed. Multiplanar axial, coronal, and sagittal T2-weighted (T2W) turbo spin-echo sequences (TSE) were performed.

For every swelling, these parameters were looked for: shape, number and size, location in relation to the urethra, location of the ostium, and signal intensity across all sequences to detect the nature of the fluid inside the swelling. On T2W sequences, ostial communication of UD was defined as a beaklike linear extension throughout the mucosa and submucosa of the urethra.

Based on MRI findings, the surgical plan is determined according to ostial number and location, complexity of the UD (septae and number of compartments), location of the UD compartments in relation to the whole circumference of the urethra. Prior to the surgery, we perform a urethroscopy to identify the ostium if possible. Once the ostium is identified, it is calibrated with a 4-Fr ureteric stent. If it fails to pass, a guide wire is introduced instead. Inverted U-shaped anterior vaginal incision is done to provide maximal exposure of the swelling. Care is taken not to perforate the swelling during dissection to help in its complete dissection. In cases of UD, the periurethral layer must be identified and incised transversely to ensure adequate exposure of the UD and its extensions around the circumference of the urethra to facilitate its complete excision.

Dissection is continued in all directions around the urethra till the UD is only connected to the urethra at the site of the communication. The connection is then cut, and the urethral defect is closed with 4–0 interrupted polyglactan sutures. In cases where extensive dissection is done with fear of possible sphincteric derangement, marsupialization is only done to prevent postoperative SUI or urethrovaginal fistula. A urethral catheter is left in place for 3–10 days according to the extent of the urethral defect.

Post-operative visits were planned as follows: 1 week, 3 months, and 6 months post-operatively, to evaluate the presence of complications and any recurrences of the masses or the symptoms.

### Statistical analysis

Statistical analysis was performed with IBM-SPSS v.17 for Windows (IBM Corp, Armonk, NY, USA). Student’s *t* test and the Mann–Whitney *U* test were performed to compare continuous parametric and nonparametric variables, as appropriate. Continuous variables were reported as %. All values in the text and tables are expressed as %.

## Results

A total of 57 patients with urethral/periurethral vaginal swellings were considered for the analysis. Patients’ demographics and characteristics are shown in Table 1.

**Table 1:** Patients’ characteristics and demographics

Patients characteristics and demographics	Number
Total number of patients	57
Mean age (in years)	43 (20–63)
Nulliparous patients	4 (7%)
Normal vaginal delivery (NVD)	48 (84.2%)
Cesarian section (C/S)	20 (35%)
Weight (in kgs)	78 (50–97)
Duration of symptoms before reaching final diagnosis (in months)	33 (1–60)
Symptoms and signs	
i. Bulge per vagina	57 (100%)
ii. Recurrent UTI (including dysuria)	28 (49%)
iii. Dyspareunia	31(54%)
iv. Obstructive urinary symptoms	10 (17.5%)
v. Splaying of urine	5 (8.7%)
vi. Storage symptoms (urgency and frequency)	39 (68.4%)
vii. Urgency urinary incontinence (UUI)	32 (56%)
viii. Stress urinary incontinence (SUI)	14 (24.5%)
ix. Postvoid dribbling	9 (15.7%)
x. Recurrent vaginal swelling	13 (22.8%)
xi. Expression of urine or pus per urethra upon compression of the vaginal swelling	27 (47.3%)

Thirteen patients (22.8%) presented with recurrent vaginal swelling after experiencing surgical intervention to remove it in other hospitals. Of these, two patients did not perform any kind of preoperative imaging, seven underwent transvaginal US only, two underwent VCUG only, and two underwent both VCUG and transvaginal US, while none underwent MRI. However, these 13 patients underwent MRI in our institute before their definitive surgery.

For the remaining 44/57 (77.2%), 34 patients underwent MRI only, seven underwent MRI and transvaginal US, and three underwent MRI and VCUG.

**Fig. 1:**
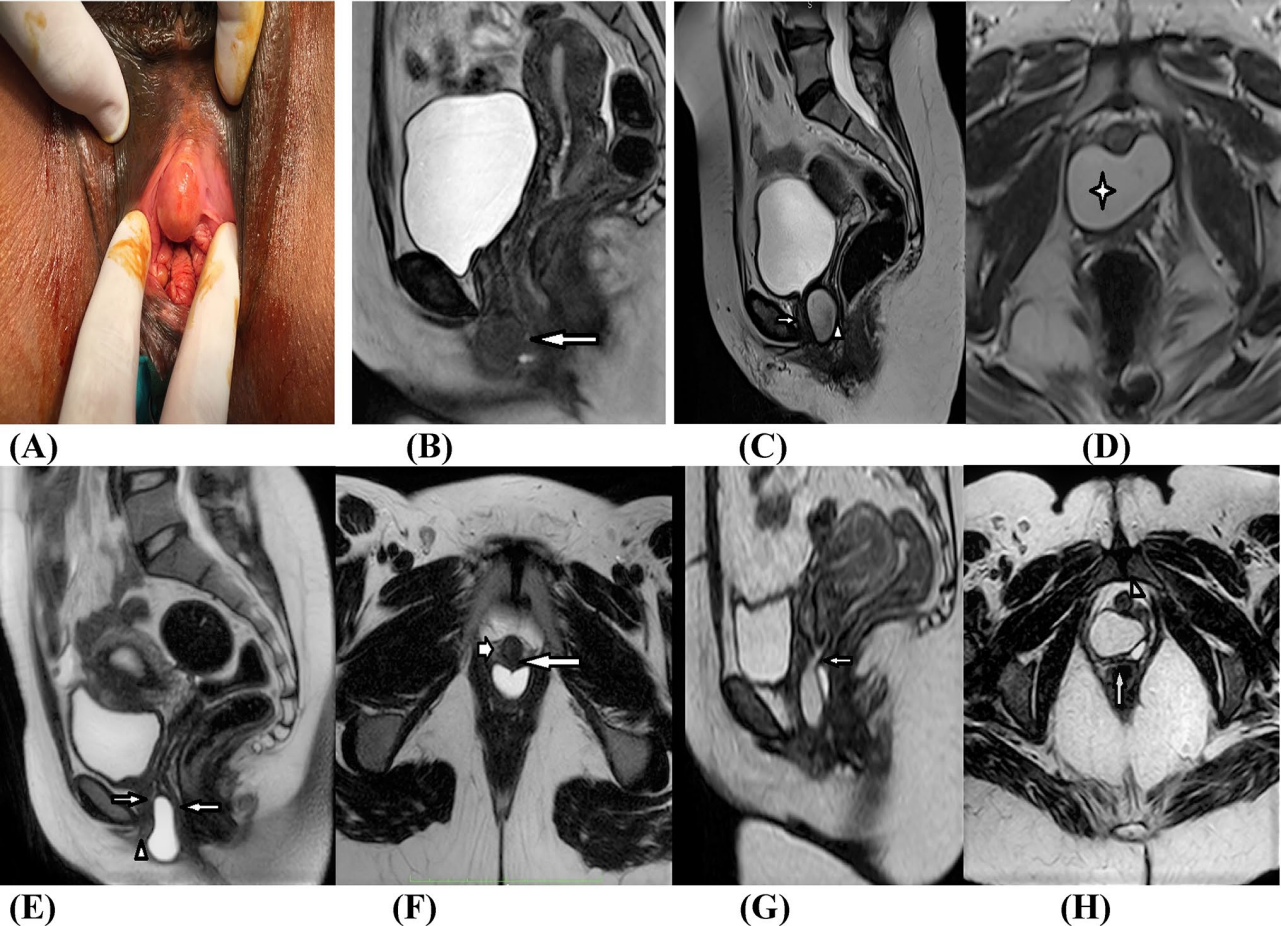
Different cystic periurethral anterior vaginal wall swellings. Skene gland cyst was evident on vaginal examination, with its characteristic juxtameatal location causing eversion of the urethral meatus (**A**). MRI T2W multiplanar sagittal (**B**) revealed globular Skene’s gland cyst (arrows in **B**) centered midline just inferior to the external urethral meatus and the symphysis pubis, and separable from the vagina, slightly effacing it anteriorly with no definite connection to the urethra. It shows signal intensity of concentrated high proteinaceous material notably T2 hypointense and T1 hyperintense. MRI T2W sagittal (**C**) and T2W axial oblique images (**D**) revealed a saddlebag cystic structure seen just postero-inferior to the bladder trigone, insinuating between the urethra (arrow in **C**) anteriorly and vagina (arrowhead in **C**) posteriorly. The lesion is seen effacing the urethra anteriorly giving the impression of partial encasement from the 3 to the 9-clock axes and pushing on the anterior vaginal wall posteriorly, with no definite connection identified. The cyst contains proteinaceous material with T2 isointense (Asterix in **D**) and T1 iso- to hyperintense. MRI T2 W sagittal (**E**) and T2 WI axial oblique (**F**) images revealed a primary vaginal cystic lesion totally contained within the lower third of the vagina, splaying both anterior and posterior vaginal walls (arrows in **E** & **F**), protruding through the introitus effacing the urethra (short arrow in **F**) and external urethral meatus (arrowhead in **E**) anteriorly with no connection identified, hence excluding urethral origin. MRI T2 W sagittal (**G**) and T2 WI axial oblique images (**H**) revealed a primary vaginal cystic lesion totally contained within the upper two thirds of the vagina and ballooning its lumen, however still with intact surrounding vaginal walls (arrow in **H**). No connection to the urethra (arrowhead in **H**) was evident. The cyst harbored clear fluid with no signs of infection. Since a linear vestigial tubular structure was identified connecting the cystic lesion to the vaginal fornix (arrow in), diagnosis of Gartner’s cyst was suggested by MRI

**Fig. 2:**
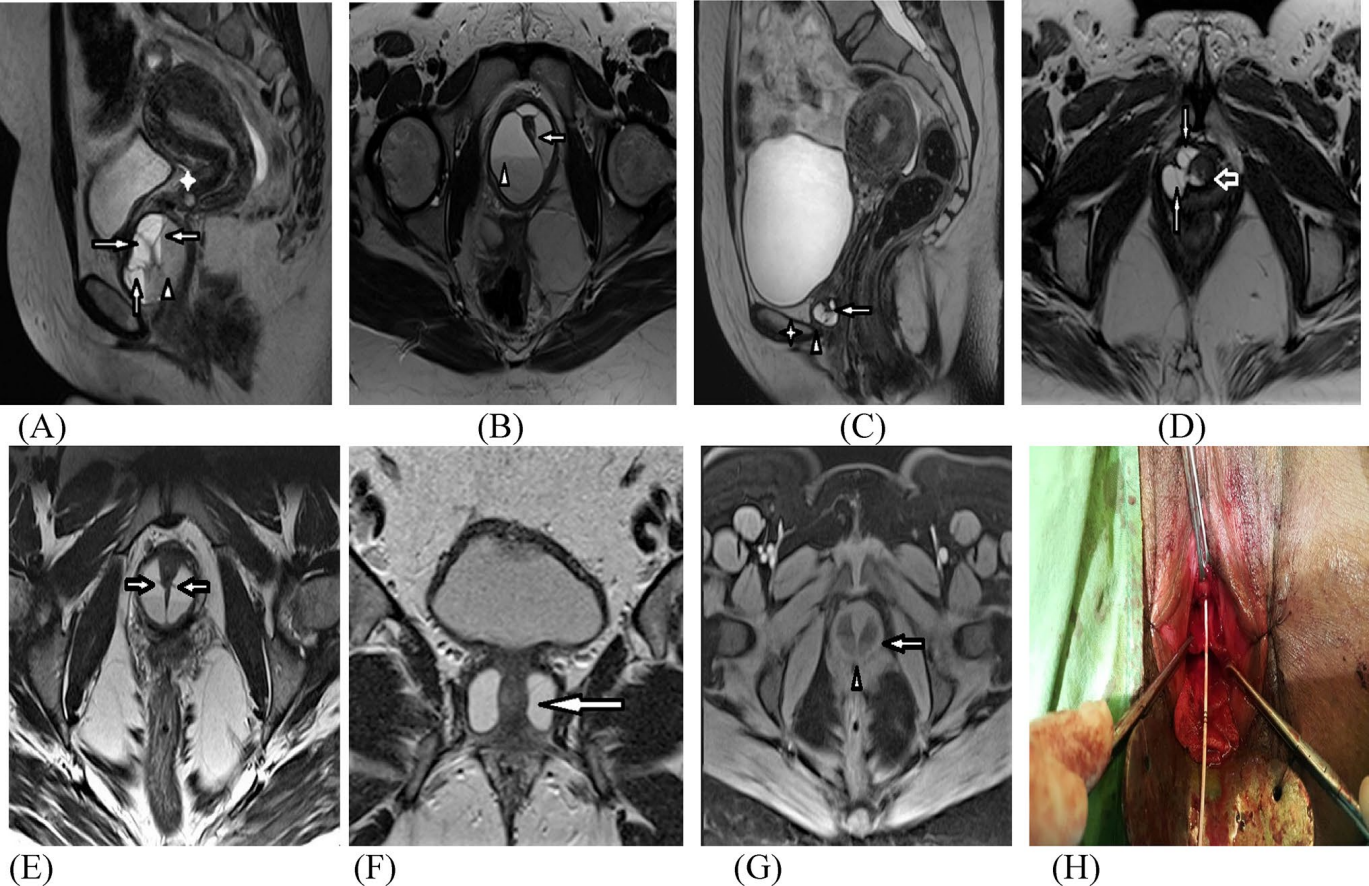
Different complex UD. MRI T2 WI sagittal (**A**) and T2 WI axial oblique (**B**) images revealed circumferential UD located posterior and above the symphysis pubis, totally encasing the urethra (**B**), with evident connection to the urethral lumen (arrow in **B**) and multiple thick septa (multiple long arrows in **A**), harboring mixed intensities of protein- urine level of debris sedimentation (arrow head in both **A** & **B**) with evident inflammatory/ infective changes. MRI T2 W sagittal (**C**) and T2 WI axial oblique (**D**) images revealed saddlebag urethral diverticulum (arrowhead in **C**) located posterior and above the symphysis pubis (Asterix on **C**), partially encasing the urethra with evident connection to the urethral lumen at the 6-clock axis (hollow arrow in **D**) and multiple thick septa (multiple long arrows in **C** & **D**), still showing homogenous T2 hyperintense signal of clear urine excluding inflammatory/infective changes. MRI T2 W multiplanar axial oblique (**E**) and coronal oblique (**F**) and T1WFS axial oblique (**G**) images revealed sizable recurrent circumferential biloculated septated urethral diverticulum located posterior and above the symphysis pubis near totally encasing the whole length of the urethra (arrow in **F**), pushing it anteriorly with evident thick central septum extending along the 6-clock axis (arrowhead in **G**) and bilateral connection to the urethral lumen (bilateral arrows in **E**), associated with additional few thin septa (bilateral arrows in **E**), harboring mixed intensities persistent on fat suppressed image, reflecting high protein-urine level of debris sedimentation (arrow in **G**), with evident inflammatory/ infective changes. After dissection and exposure of the complex UD, both loculi were identified with midline septum in between. The ostium was calibrated with a ureteric catheter via cystoscopy performed prior to the surgery (**H**)

MRI identified eight (14.04%) skene gland cysts, four (7.02%) Mullerian cysts, two (3.51%) Gartner cysts, and six (10.53%) inclusion dermoid cysts (Fig. 1). This was further confirmed after surgical intervention and final pathology analysis. 37/57 (64.9%) patients received the diagnosis of UD. MRI was able to identify the ostium in 34/37 cases (91.9%). Regarding the location, 9 UD were distal in location, 22 were related to the mid-urethra, and 6 were proximal. Simple UD was identified in 5/37 (13.5%) cases. MRI revealed 8/37 (21.6%) cases of circumferential UD encasing the urethra, with 4/8 being septated (Fig. 2). Twenty-four (64.8%) saddlebag UD (Fig. 2) were identified revealing septations in 5/24, with a single UD being anteriorly located. Two patients were misdiagnosed as cystocele, hence underwent traditional colporrhaphy by a gynecologist, and they presented to us by recurrent vaginal swelling. MRI showed biloculated septated circumferential UD with thickened walls (Fig. 2).

Cystourethroscopy was only successful to identify the ostium of UD in 29/37 cases (78.4%). Complete excision of the UD was amenable in 32 cases (86.4%). The remaining 5 cases had marsupialization of the cyst only due to difficult dissection and fear of extensive urethral defect. Perioperative and postoperative complications were minor. Nine patients (9/57) had minimal intraoperative bleeding from the vaginal dissection, which was controlled intraoperatively, and a vaginal pack was left for 24 h. Five patients (5/57) had UTI postoperatively. We did not experience a single case of recurrent vaginal swelling after our surgical intervention in our institute. Mean duration of follow-up of our patients was 35 months (with a range of 6–62 months).

## Discussion

Female urethral and periurethral cystic swellings represent a great challenge for the urogynecologists. Diagnosis of these lesions is difficult due to short female urethra, intermingling urogynecological symptoms, rarity of these lesions, and the anatomical location of these lesions in relation to the complex pelvic structures [6, 8].

All these obstacles reflect on the duration to reach accurate final diagnosis and type and number of preoperative radiological imaging. The mean duration to reach accurate diagnosis is variable in the literature, ranging from 2 to 6 years with a mean of 5.2 years [7, 9]. In our study, the mean duration was 2.75 years. We believe that the mean duration in our study is shorter, because our center is a tertiary referral center.

These lesions usually present with a wide range of overlapping symptoms. Symptoms include vaginal bulge, classical 3Ds of dyspareunia (12–14%) [3], dysuria (30–50%) and postvoid dribbling (10–30%), recurrent UTI (40%) [10], urinary incontinence (32%) [11], expression of pus or urine per urethra upon compression (25%) [12], and obstructive urinary symptoms (4%) [10]. Although the triad of classical 3Ds is pathognomonic, it is collectively present in only 5% of the cases. [13]. About 20% of patients with cystic urethral/periurethral swellings are asymptomatic [7].

Radiological modalities available for accurate detection of the nature of these cystic lesions are variable. VCUG was frequently used in the past to delineate UD. It is considered hazardous due to radiation exposure and need for catheterization with risk of UTI. It is also solely dependent on voiding of the patient. In acutely inflamed UD, VCUG will not opacify them and thus will miss their diagnosis [14]. Sensitivity of VCUG in detection of UD is only 65% [15]. DBU is an ancient technique for diagnosis of UD, with better diagnostic validity than VCUG (75%) [16]. It requires a designated catheter and experienced radiologist to perform. Likewise, it carries hazards of radiation and contrast exposure [17]. Both VCUG and DBU cannot diagnose other non-communicating urethral/periurethral cystic swellings.

Different ultrasound techniques are also available. It is non-invasive without risk of radiation exposure or contrast injection. It also has higher sensitivity than VCUG and DBU, but it is operator-dependent and cannot be interpreted by the surgeons [14].

MRI is considered the gold standard imaging modality. It does not require catheterization, no contrast injection, and no radiation exposure, and is not dependent on voiding of the patient [18]. It allows multiplanar scanning and visualization of the complex pelvic structures and muscles, with excellent demarcation of the urethral anatomy. It permits visualization of lesions outside the urethra that may present with similar clinical symptoms [16, 19, 20]. It provides spatial data that can aid in preoperative surgical planning according to the complexity of the lesion [18, 21, 22].

These cystic swellings include UD, which is the most common lesion representing 84% of all periurethral lesions [1, 6], Skene gland cysts, Gartner duct cysts, Mullerian remnant cysts, and inclusion dermoid cysts. MRI can accurately differentiate between cystic and solid lesions, providing a roadmap before surgery [6]. Distinction between UD and other periurethral cysts is challenging, but the differentiation is crucial because surgical technique for the two categories differs. It is ideal to completely remove all the lesion, which is not amenable in all cases. However, UD requires complete excision to avoid recurrence and rare incidence of malignant transformation in 1–6% [1, 7, 23]. Circumferential UD may even require urethral reconstruction according to its location and proximity to the bladder neck [19].

UD arise from the posterolateral aspect of mid-urethra. Classically, non-complicated UD exhibit signal of clear urine which is hypointense on T1W images and hyperintense on T2W image. If they are complicated by blood or protein content, they display variable iso- to hyperintensity on T1W sequences, and variable hypointensity on T2W images. Inflamed/infected UD show heterogeneous signal intensity on T1W images, and marked hyperintensity on T2W fluid–fluid level [24, 25]. Ostial connection identification is the cardinal sign of diagnosis of UD on MRI.

Gartner duct cysts are embryological retention cysts arising from Wolffian duct remnants, anywhere from the mesosalpinx through the broad ligament to the cervix and anterolateral upper third of vagina, above the level of symphysis pubis [26]. A classical discriminating point from UD is lack of urethral connection, with no urethral displacement or deformity. Sometimes, the vestigial connection to the vaginal wall could be identified. Furthermore, they can be associated with other mesonephric duct anomalies such as unilateral renal agenesis, renal hypoplasia, or ectopic ureter [24, 27]. Müllerian cysts are embryologic remnants of the Müllerian duct, being present in the anterolateral vaginal wall. Differentiation of Gartner and Mullerian cysts is a challenge; however, it is of no clinical or surgical importance [6].

On MRI, Skene gland cysts are teardrop like cysts, typically located juxtameatal, being lateral to the external urethral meatus and inferior to the pubic symphysis. They secrete mucus-containing fluids which is protein-based, hence giving the protein signal of iso- to hyperintensity on T1W sequences. This becomes highest when obstructed, turning the retained secretions into cheesy like material [28].

Epidermal vaginal inclusion cysts are situated at sites of prior surgery or vaginal trauma. They are located mainly at posterior vaginal walls. They demonstrate intermediate signal intensity on T1W images [6]. In our series, MRI correctly diagnosed the nature of these lesions prior to surgery. This was confirmed after surgical intervention and pathology analysis. Moreover, it provided precise anatomical configuration and complexity of UD, with detailed identification of the location of the UD in relation to the urethra, and the ostial communication between it and the urethra.

Recurrent UD is reported in up to 40% after transvaginal excision of UD. Risk factors for UD recurrence are multiplicity, proximal location, and complex configuration of UD. When MRI is not utilized, the risk of incomplete removal of UD is high due to underdiagnosis of the complexity and the extent of the UD [29]. Repeat urethral diverticulectomy imposes a nightmare to every urogynecologist due to disturbed anatomy and presence of extensive fibrosis from previous surgery and recurrent infections, with resultant inability to enter the proper surgical plane [7].

The 13 patients presenting with recurrent vaginal swellings were previously evaluated with imaging modalities other than MRI. They underwent MRI in our institute which revealed complex UD (saddlebag or circumferential with septations) in all cases. We believe if these patients had performed MRI prior to their initial surgery, the complexity of the UD would have been evident, and appropriate preoperative surgical planning would have been beneficial in achieving complete excision and avoidance of recurrence. In our institute, we perform MRI as a standalone diagnostic imaging modality in all vaginal swellings. MRI is a mandatory tool for proper preoperative surgical planning to understand the complexity of the lesions and precise anatomy of the urethra in relation to the vaginal swelling.

In our series, UD was identified in 37 cases. Five were simple diverticula, while 32 cases were of complex anatomical configuration. MRI was helpful in precise identification of all complex lesions as confirmed during the surgery. This allowed complete excision of the entire UD. All patients were followed-up with a mean duration of 35 months, without evidence of recurrence. This signifies the importance of preoperative MRI to ensure surgical success, especially in complex UD.

One of the limitations of the utilization of MRI in every suspected case is the cost. However, if we consider its high sensitivity in detection of these lesions, we will avoid multiple preoperative imaging modalities which will collectively add up to a probably equivalent cost and needless unplanned surgical interventions, with a high chance of failure and recurrence. This will make MRI a cost-effective tool in diagnosis of these lesions with proper surgical planning.

### Strengths and limitations of our study

To our knowledge, this is the largest series of cystic urethral and periurethral swellings in the literature, with longest follow-up period. All the cases were operated by the same surgical team, and the preoperative MRI was interpreted by the same radiologist, who was blinded to the clinical data. Limitations of our study include the retrospective nature with subsequent lack of statistical data and the fact that we did not perform postoperative MRI to ensure complete excision of the lesions; instead, we depended on the clinical symptoms and examination.

## Conclusion

Periurethral and urethral cystic swellings are uncommon lesions, with overlapping urogynecological symptoms. Identification of the nature and anatomical complexity of these lesions, especially complex UD, is of crucial importance. MRI as a standalone imaging technique is mandatory for diagnosis of all these cystic lesions, as it offers the most accurate modality for delineation of these lesions. This will aid in the preoperative surgical planning, aiming to avoid recurrences and improve surgical outcomes.
